# Hepatitis D Virus Infection of Mice Expressing Human Sodium Taurocholate Co-transporting Polypeptide

**DOI:** 10.1371/journal.ppat.1004840

**Published:** 2015-04-22

**Authors:** Wenhui He, Bijie Ren, Fengfeng Mao, Zhiyi Jing, Yunfei Li, Yang Liu, Bo Peng, Huan Yan, Yonghe Qi, Yinyan Sun, Ju-Tao Guo, Jianhua Sui, Fengchao Wang, Wenhui Li

**Affiliations:** 1 Graduate Program in Chinese Academy of Medical Sciences and Peking Union Medical College, Beijing, China; 2 National Institute of Biological Sciences, Zhongguancun Life Science Park, Changping, Beijing, China; 3 Graduate School of Beijing Normal University, Beijing, China; 4 Drexel Institute for Biotechnology and Virology Research, Drexel University College of Medicine, Doylestown, Pennsylvania, United States of America; University of California, San Diego, UNITED STATES

## Abstract

Hepatitis D virus (HDV) is the smallest virus known to infect human. About 15 million people worldwide are infected by HDV among those 240 million infected by its helper hepatitis B virus (HBV). Viral hepatitis D is considered as one of the most severe forms of human viral hepatitis. No specific antivirals are currently available to treat HDV infection and antivirals against HBV do not ameliorate hepatitis D. Liver sodium taurocholate co-transporting polypeptide (NTCP) was recently identified as a common entry receptor for HDV and HBV in cell cultures. Here we show HDV can infect mice expressing human NTCP (hNTCP-Tg). Antibodies against critical regions of HBV envelope proteins blocked HDV infection in the hNTCP-Tg mice. The infection was acute yet HDV genome replication occurred efficiently, evident by the presence of antigenome RNA and edited RNA species specifying large delta antigen in the livers of infected mice. The resolution of HDV infection appears not dependent on adaptive immune response, but might be facilitated by innate immunity. Liver RNA-seq analyses of HDV infected hNTCP-Tg and type I interferon receptor 1 (*IFNα/βR1*) null hNTCP-Tg mice indicated that in addition to induction of type I IFN response, HDV infection was also associated with up-regulation of novel cellular genes that may modulate HDV infection. Our work has thus proved the concept that NTCP is a functional receptor for HDV infection *in vivo* and established a convenient small animal model for investigation of HDV pathogenesis and evaluation of antiviral therapeutics against the early steps of infection for this important human pathogen.

## Author Summary

Currently 15 million people worldwide are infected by hepatitis D virus (HDV). HDV is the smallest virus known to infect human. With co-infection of its helper hepatitis B virus (HBV), viral hepatitis D is considered as the most severe form of viral hepatitis. No specific anti-HDV drugs are available; antivirals against HBV do not ameliorate hepatitis D. We report mice expressing a human bile acids transporter sodium taurocholate co-transporting polypeptide (NTCP) in the liver support HDV infection, providing a useful model for studying antivirals against HDV and understanding how the simplest virus interacts with a host *in vivo*. Our transcriptome analyses of livers of infected mice have unveiled interaction landscape of HDV and the hosts, laying a foundation for further studies.

## Introduction

Hepatitis D virus (HDV) is the smallest virus known to infect humans with a single—stranded, circular RNA genome of about 1.7 kilobases in length. It is enveloped by surface proteins from its helper hepatitis B virus (HBV) and undergoes a unique replication cycle via an intermediate, antigenomic RNA [1,2]. Prevalence of HDV infection remains high in many areas around the world despite the implementation of vaccine programs against HBV. Currently 15 million people are infected by HDV among the 240 million chronic HBV carriers. Viral hepatitis D is considered as one of the most severe forms of human viral hepatitis. However, there are no specific antivirals available for clinical treatment of the infection and antiviral therapies against HBV do not ameliorate hepatitis D. The mechanisms that determine whether an individual clears HDV spontaneously or becomes chronically infected are unclear [3,4]. Understanding HDV infection and developing antivirals against HDV have been hampered by the lack of reliable cell culture systems and convenient small animal models susceptible for efficient HDV infection.

Sodium taurocholate co—transporting polypeptide (NTCP), a multiple transmembrane transporter predominantly expressed in the liver [5], was recently identified as a common entry receptor for HDV and HBV [6]. This finding has enabled cell culture systems that support HDV and HBV infection *in vitro*. For instance, exogenous expression of human NTCP (hNTCP) rendered HDV infection of multiple mammalian cell lines, while successful HBV infection has only been achieved in hNTCP—expressing human hepatoma cells [6–9]. It is reasonable to speculate that additional human hepatocyte—specific factors are required for HBV infection of mice, however, transgenic expression of hNTCP may confer susceptibility of mouse hepatocytes to *de novo* HDV infection, which may provide a much—needed convenient small animal model for investigation of HDV pathogenesis and evaluation of antiviral drugs against HDV *in vivo*. In addition, as no other virus is simpler than HDV yet still can infect mammals, studying HDV infection in a susceptible mouse model may also help to illuminate how an animal reacts to the invading of the smallest pathogen.

We report herein that transgenic mice expressing hNTCP in hepatocytes, designated as hNTCP—Tg, support *de novo* HDV infection. Active HDV genome replication in the livers of infected mice was demonstrated by the presence of antigenomic RNA and edited RNA species. Infection kinetic studies revealed that HDV infection of hNTCP-Tg mice was acute and age—dependent. The infection was efficiently blocked by monoclonal antibodies specifically recognizing the critical regions of HBV envelope proteins. In our efforts toward unraveling the mechanism underlying the resolution of HDV infection in the hNTCP-Tg mice, we obtained evidence suggesting that adaptive immunity was not required for the clearance of HDV infection in the mouse model. Instead, HDV infection of hNTCP-Tg mice induced a type-I interferon (IFN) response that might have contributed to the suppression of HDV replication. Intriguingly, HDV infection could also be efficiently cleared in hNTCP-Tg type I interferon receptor 1 (*IFNα/βR1*) null mice. RNA—seq analyses of liver transcriptome of the HDV infected hNTCP-Tg and hNTCP-Tg/*IFNα/βR1* ^-/-^ mice revealed that, in addition to known interferon—stimulated genes (ISGs), HDV infection was also associated with up—regulation of novel cellular genes yet uncharacterized for antiviral activity.

## Results

### Human NTCP transgenic C57BL/6 mice

**Fig 1:**
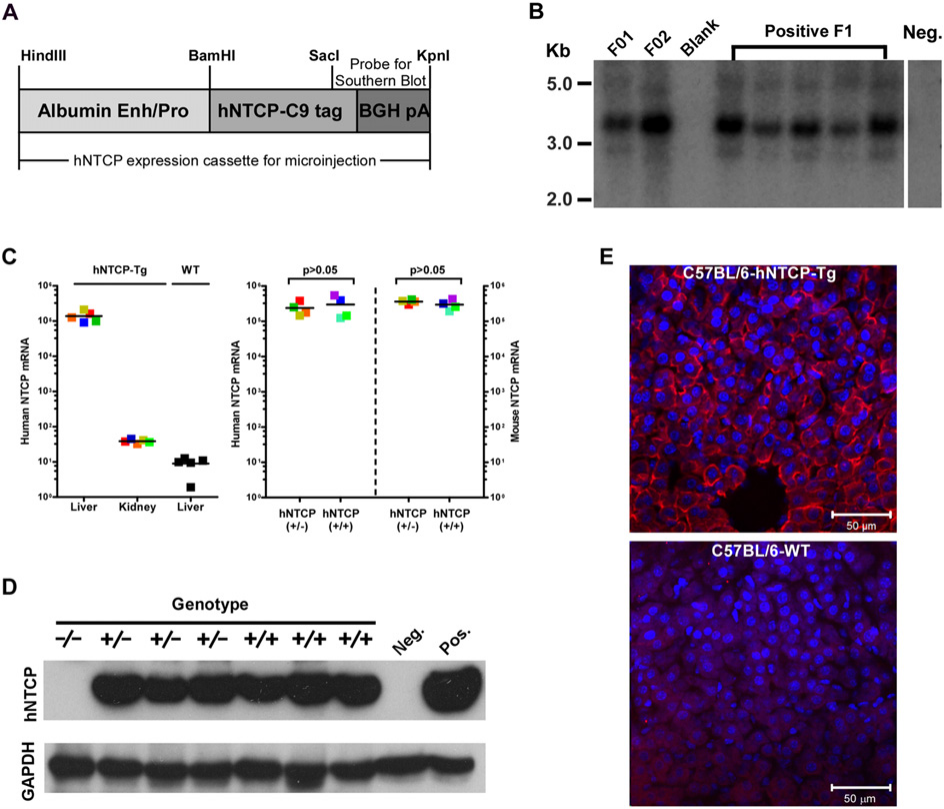
Human NTCP transgenic (hNTCP-Tg) C57BL/6 mice. **(A)** Schematic diagram of human NTCP transgene introduced into mouse genome. Expression of hNTCP was controlled by a mouse albumin enhancer/promoter with a Bovine Growth Hormone (BGH) polyadenylation signal. The human NTCP gene was fused with a tag (C9) at its C—terminus (hNTCP-C9). The position of the probe for Southern blot analysis of human NTCP transgene was shown. **(B)** Southern blot analysis of the hNTCP transgene. Genomic DNA from the hNTCP-Tg or a wild—type (WT) mouse was digested with *Bam* HI. 5 μg of digested genomic DNA was separated by agarose gel electrophoresis and analyzed by Southern blot hybridization with a [α-32P] dCTP—labeled probe containing a 428bp chimeric fragment from 3’ hNTCP and BGH poly A region. The F1 offspring positive in PCR screening were examined for germline transmission of hNTCP, indicated by the presence of a 3.67 kb band, the F1 mice were from Founder 1 (F01). **(C–E)** Expression of human NTCP in the hNTCP-Tg mice. **(C)** Human NTCP mRNA level was assessed with quantitative realtime PCR after reverse transcription (qRT-PCR) in the transgenic mice (n = 5) or wild—type mice (n = 5) (*left*). mRNA level of human NTCP or mouse Ntcp in the hNTCP-Tg homozygotes (n = 4) or heterozygotes (n = 4) mice was assessed by qRT-PCR (*right*). The mRNA copy numbers per 20 ng total liver RNA were presented, the detecting limit was ~10 copies per 20 ng liver RNA. The mRNA level of mNtcp and hNTCP was comparable in hNTCP-Tg mice. Squares with the same color represent data—points from the same mouse; bars indicate the median of each group. Statistical significance was calculated by Mann—Whitney—Wilcoxon Test. **(D)** Western blot analysis of hNTCP-C9 transgene in total 30 μg liver lysate. Expression of hNTCP transgene was detected by a monoclonal antibody (1D4) that specifically recognizes the C—terminal tag (C9) of human NTCP transgene. Mouse GAPDH was used as a loading control. **(E)** Immunofluorescence staining of liver from an hNTCP-Tg mouse or a wild—type littermate. Expression of the hNTCP transgene was detected by staining the C9 tag in red; nuclei were stained with DAPI in blue.

We and others previously demonstrated that HDV infection is restricted by murine Ntcp in cell cultures, various mammalian cells complemented with human— but not mouse NTCP supported HDV infection [7,8]. To generate a mouse model for HDV infection, we created C57BL/6 mouse lines expressing hNTCP with a C—terminal tag (C9), driven by a mouse albumin enhancer/promoter (Fig 1A). Mice were screened for the transgene by real—time PCR with primers specific for the human NTCP, and the expression levels of hNTCP mRNA in the liver were examined by real—time PCR after reverse transcription (qRT-PCR). One hNTCP transgenic (hNTCP-Tg) C57BL/6 mouse line that was confirmed for germline transmission of the hNTCP transgene by Southern blot analysis (Fig 1B) and expression of high level human NTCP mRNA in liver (Fig 1C—*left*) was selected for breeding and subsequent experiments. The expression level of hNTCP transgene was similar to that of the endogenous murine Ntcp (Fig 1C-*right*). There was no significant difference in the expression of hNTCP transgene between the homozygotes and heterozygotes at both mRNA level quantified by qRT-PCR (Fig 1C—*right*) and protein level examined by Western blot using antibodies against the C9 tag (Fig 1D). Immunofluorescent staining of liver sections with a C9—tag specific antibody showed hNTCP only expressed in the hepatocytes of hNTCP transgenic mice (Fig 1E).

### HDV infects human NTCP transgenic mice *in vivo*

**Fig 2:**
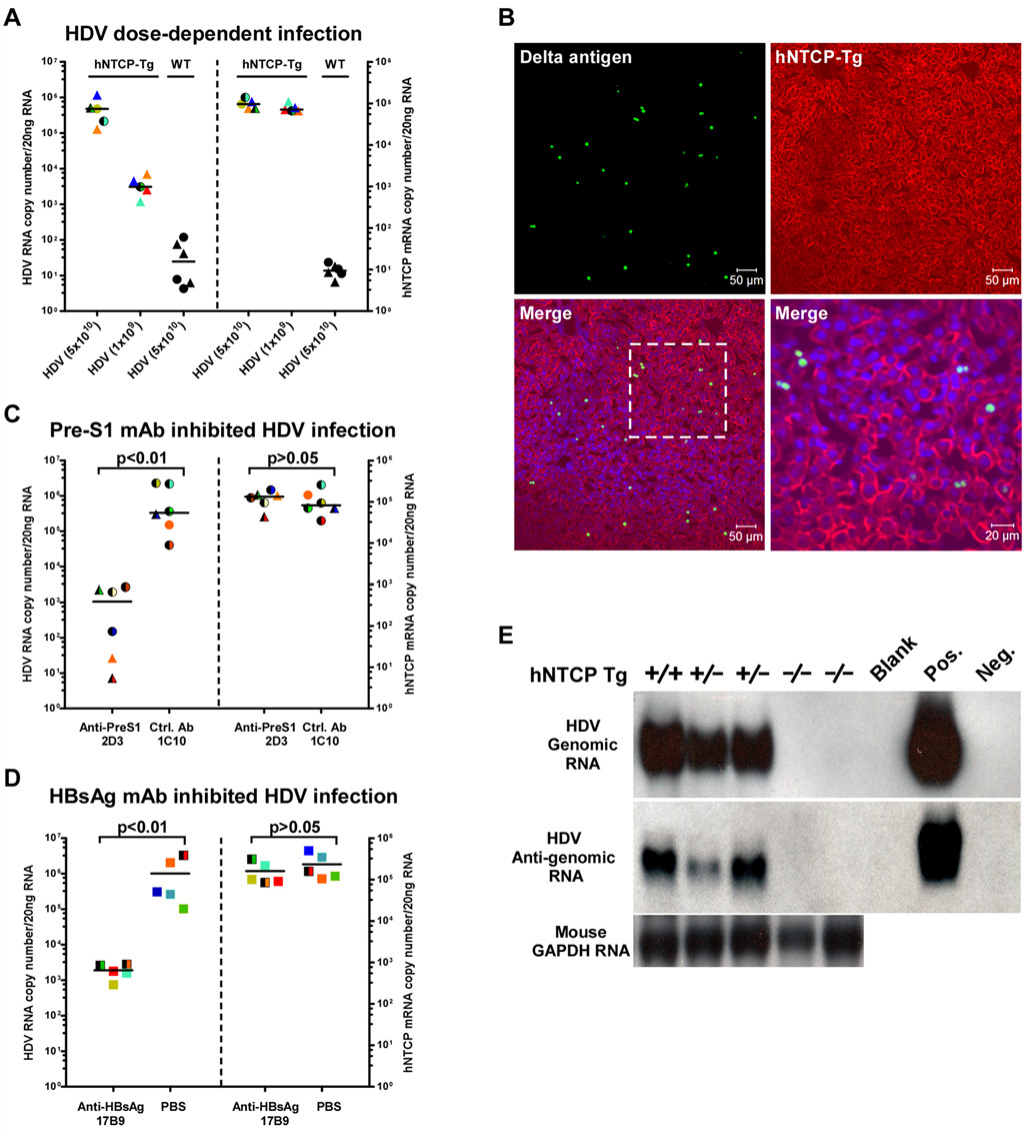
HDV infects human NTCP transgenic mice *in vivo*. The hNTCP transgenic C57BL/6 mice were inoculated with HDV by intraperitoneal (i.p.) injection on day 9 after birth. **(A)** hNTCP-Tg or wild—type littermates were inoculated with indicated genome equivalents (GEq) HDV. Mice were sacrificed 6 days after viral inoculation. RNA levels of HDV and human NTCP was determined by qRT-PCR. **(B)** Immunofluorescence staining of HDV delta antigens in liver. Photos taken from liver sections of a mouse challenged with 3.3×10^10^ GEq HDV were shown. The human NTCP transgene was stained by the C9 tag in red; HDV delta antigens in green; nuclei in blue. **(C)** qRT-PCR analysis of HDV RNA levels in the hNTCP-Tg mice challenged with 6.7×10^10^ GEq HDV. A mouse monoclonal antibodies (mAb) 2D3 targeting to the pre-S1 region of HBV L protein was injected i.p. (10mg/kg) one hour prior to the viral challenge, a mcAb 1C10 (10mg/kg) recognizing the core protein of HBV was used as a control. RNA copy numbers of HDV and human NTCP were determined on day 6 post infection. 2D3 blocked more than 95% HDV infection in hNTCP-Tg mice; the hNTCP mRNA level was similar between the two groups after the infection. **(D)** qRT-PCR analysis of the levels of HDV RNA in the mice inoculated with 4.3×10^10^ GEq HDV. A monoclonal antibody17B9 targeting to the S region of HBV envelop proteins was injected i.p. (10mg/kg) one hour before the viral challenge, PBS was used as a control. RNA copy numbers of HDV and human NTCP were determined on day 6 post infection. **(E)** Northern blot analysis of HDV genome and antigenome RNA. Result from individual mouse inoculated with 5×10^10^ GEq HDV was shown. Mouse GAPDH RNA was used as RNA loading control. RNA from HepG2-NTCP cells infected or not by HDV was used as positive or negative control. For panels (A, C, D), data—points from individual mouse were shown in the same color and pattern. The detecting limit for HDV RNA was 10–100 copies per 20 ng liver total RNA in this and other HDV *in vivo* infection experiments. hNTCP-Tg homozygotes in mono—color, heterozygotes in two—color; male in triangle, female in circle, gender not determined in square; bars indicate the median of each group. Statistical significance was calculated by Mann—Whitney—Wilcoxon Test.

Homozygous and heterozygous hNTCP-Tg C57BL/6 mice were both tested for their susceptibility to HDV infection. The viral infection efficiency in hNTCP-Tg mice of 9–10 days old (n = 10) correlated with the dose of inoculating virus and was independent of the hNTCP transgene homozygosity or gender of the transgenic mice (Fig 2A). At the highest HDV dose (5×10^10^ genome equivalents, GEq) tested, no infection was observed in the wild—type littermates (n = 6) that shared the same genetic background, microbiota and environment with the hNTCP-Tg mice. In the hNTCP-Tg mice, approximately 3% hepatocyte was being infected as examined by immunofluorescent staining positive for HDV delta antigens and the infection occurred in randomly scattered hepatocytes (Fig 2B). No significant liver histopathological changes were observed in the infected mice. There was only modest apoptosis, which was about 0.8% of the total cells, in the liver of HDV infected mice (S1 Fig). The HDV infection of hNTCP-Tg mice could be efficiently blocked by monoclonal antibody 2D3 specifically recognizing the pre-S1 domain of HBV large envelope protein (n = 6) [6], but not a control antibody (1C10) recognizing the core protein of HBV (n = 6) (Fig 2C). Similarly, a monoclonal antibody 17B9 targeting the S domain of the HBV envelope [6] that presumably attaches with the heparan sulfate proteoglycan on hepatocytes [10] also greatly reduced HDV infection in the mice (n = 5) (Fig 2D). These results show that HDV infection of hNTCP-Tg mice depends on both the pre-S1 and the S region of HBV envelop proteins, and suggest these animals are useful for evaluating inhibitors against HDV entry. HDV undergoes a unique replication cycle *via* an intermediate, antigenomic RNA [11]. In the hNTCP-Tg but not wild—type littermates, both genomic and antigenomic HDV RNA were readily detectable by Northern blot analysis (Fig 2E), indicating HDV effectively replicated in the hNTCP-Tg mice.

We next tested the susceptibility of the hNTCP-Tg mice to HDV infection at different age. Interestingly, while challenge of hNTCP-Tg mice younger than 17 days by intraperitoneal (i.p.) injection resulted in marked HDV infection, as indicated by the presence of approximately 1000 copies of HDV RNAs per cell (~10^6^ copies/20ng liver total RNA) at 9 days post infection in the livers of mice (S2A Fig), challenge of the transgenic mice older than 4 weeks with HDV failed to establish effective infection (S2B and S2C Fig), although these mice efficiently expressed hNTCP in the livers regardless of their genotype of being homozygote or heterozygote of the transgene. Together these results demonstrate that hNTCP transgenic mice support HDV entry and RNA replication in hepatocytes *in vivo* in an age—dependent manner.

### Kinetics of HDV infection in hNTCP-Tg, or hNTCP-Tg with Prkdc mutation (SCID) mice

**Fig 3:**
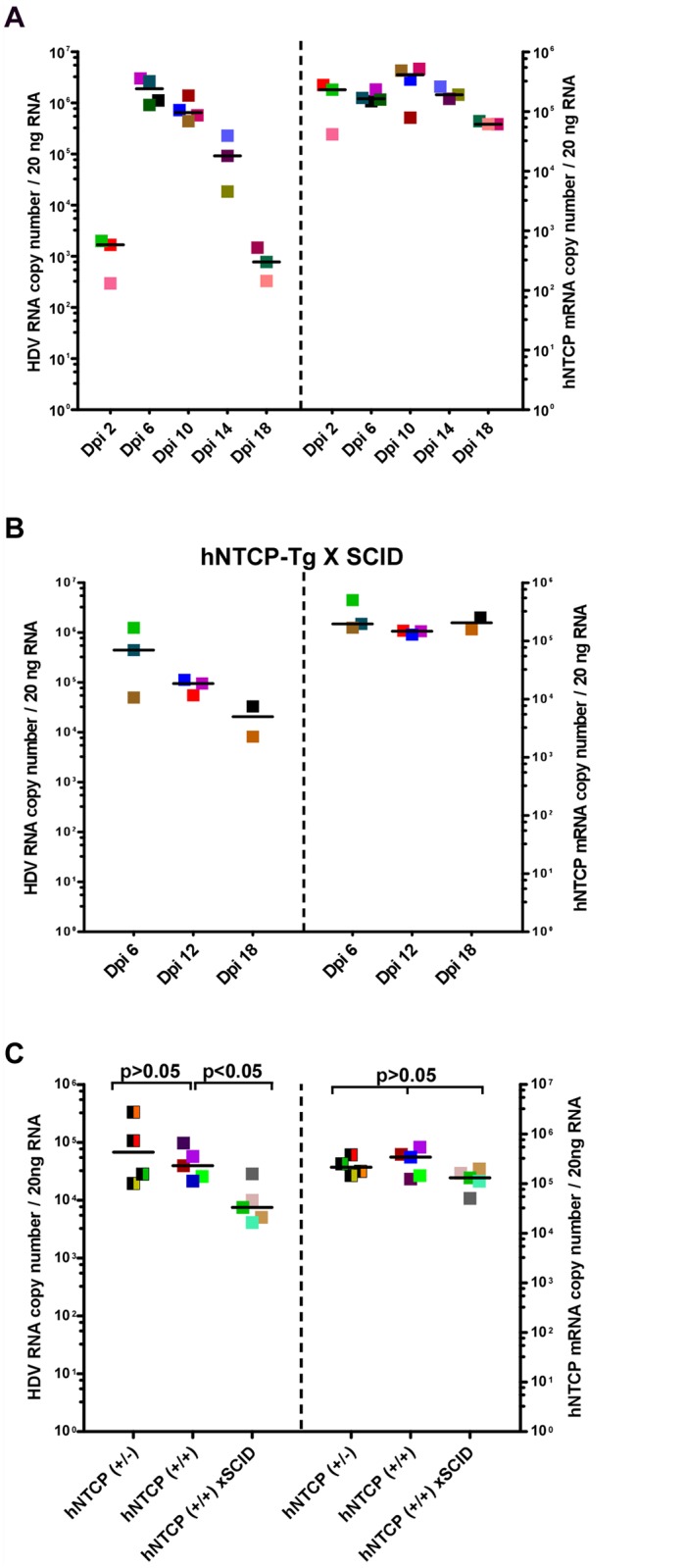
Clearance of HDV in hNTCP-Tg mice or hNTCP-Tg with Prkdc mutation (SCID) mice. (**A**) hNTCP-Tg homozygotes were i.p. inoculated with 1×10^10^ GEq of HDV 9 days after birth and sacrificed on indicated days post infection (Dpi). RNA levels of HDV and hNTCP transgene expression were determined by qRT-PCR. (**B**) hNTCP-Tg homozygotes with homozygous *Prkdc* mutation (SCID) were i.p. inoculated with 5.3×10^10^ GEq of HDV 9 days after birth, the mice were sacrificed on indicated days post infection. RNA levels of HDV and hNTCP transgene were determined by qRT-PCR. **(C)** Side—by—side experiment for direct comparison of HDV infection in the mice bearing human NTCP. Heterozygous or homozygous hNTCP-Tg mice, or hNTCP-Tg homozygotes with homozygous *Prkdc* mutation (SCID) as indicated, were i.p. inoculated with 1.3×10^9^ GEq of HDV 9 days after birth, the mice were sacrificed on day 6 post infection. RNA levels of HDV and hNTCP transgene expression were determined by qRT-PCR. For panels (A–C), results from three individual mice were shown in the same color and pattern; bars indicate the median of each group. Statistical significance was calculated by Mann—Whitney—Wilcoxon Test.

Because HDV infection of hNTCP-Tg mice should only result in a single—round infection of hepatocytes, it is interesting to know how the host immune system responds to the virus infection. In addressing this question, we first determined the kinetics of HDV replication in the liver of transgenic mice. hNTCP-Tg mice were infected by about 1×10^10^ GEq of HDV at 9 day after birth and sacrificed on 2, 6, 10, 14, and 18 days post infection. Intrahepatic HDV RNA reached peak level around 6 day post infection and then declined from the peak by approximately 1000 fold within next 12 days (Fig 3A). These observations suggest that HDV infection of the hNTCP-Tg mice is transient.

To explore host factors responsible for controlling the HDV infection *in vivo*, we first investigated the role of adaptive immunity. Specifically, a hNTCP-Tg *scid* mouse line was established by crossing hNTCP-Tg with adaptive immunity deficient *Prkdc* ^*scid*^ mice which bear a premature stop codon in the *Prkdc* gene whereby the differentiation of lymphoid cells was disrupted in these mice [12]. Similar to that observed in hNTCP-Tg mice, HDV infection was cleared rapidly in hNTCP-Tg mice with a homozygous *Prkdc* gene mutation (hNTCP / *Prkdc* ^*scid*^) (n = 8) (Fig 3B), indicating that adaptive immunity is dispensable for viral clearance in these mice. Interestingly, on day 6 after viral inoculation, intrahepatic HDV RNA level in the hNTCP^+/+^/*Prkdc* ^*scid*^ mice was significantly lower than that of the hNTCP^+/+^ mice (Fig 3C); perhaps a higher level of innate immunity activity in the *Prkdc* ^*scid*^ mice may have contributed to the rapid HDV clearance at early time [13,14].

### Transcriptome analysis of HDV infected livers from hNTCP-Tg and interferon α/βreceptor 1 (IFNα/βR1 ^-/-^) deficient hNTCP-Tg mice

**Fig 4:**
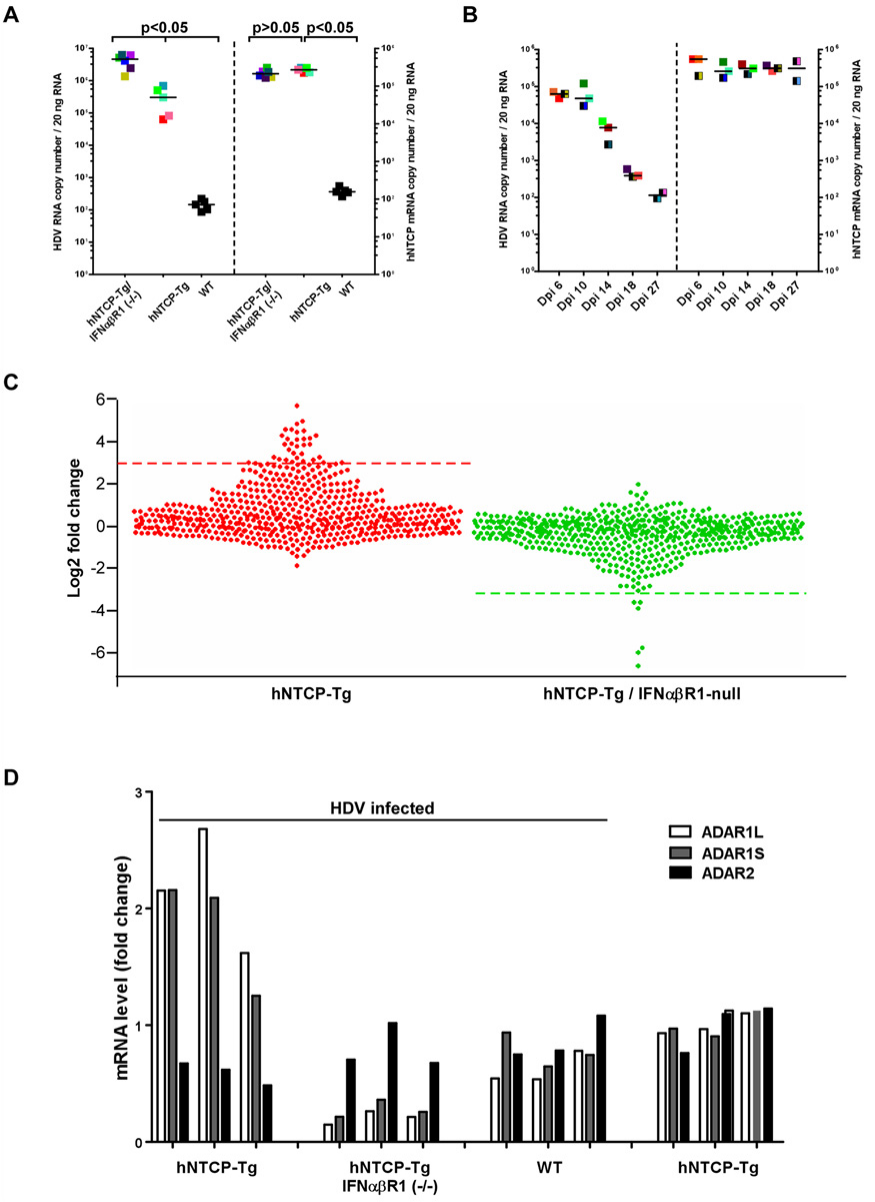
Human NTCP transgenic mice homozygous for IFNα/βR1-/- cleared HDV infection. **(A)** hNTCP-Tg mice, hNTCP-Tg *IFNα/βR1* ^-/-^ mice or wild—type C57BL/6 mice were i.p. inoculated with 4.8×10^10^ GEq HDV 9 days after birth, the mice were sacrificed on day 6 post infection. RNA levels of HDV and hNTCP transgene were determined by qRT-PCR. Results from individual mouse were shown in the same color and pattern; All hNTCP-Tg mice were homozygotes of hNTCP transgene and were in mono—color; bars indicate the median of each group. Statistical significance was calculated by Mann—Whitney—Wilcoxon Test. **(B)** *IFNα/βR1* ^-/-^ mice with hNTCP transgene were i.p. inoculated with 7.9×10^9^ GEq HDV 9 days after birth, the mice were sacrificed at indicated days after infection. RNA levels of HDV and hNTCP transgene were determined by qRT-PCR. Results from individual mouse were shown in the same color and pattern. All mice were null of IFNα/βR1 (*IFNα/βR1* ^-/-^); hNTCP-Tg homozygotes in one—color, heterozygotes in two—color; bars indicates the median of each group. **(C)** Dot plots of ISGs expression fold change in hNTCP-Tg, or hNTCP-Tg/*IFNα/βR1* ^-/-^ mice in comparison to wild—type mice. All mice were inoculated with 1.6×10^10^ GEq HDV on day 9 after birth and sacrificed at day 6 post infection. Dots above the red line *(left)* or below the green line *(right)* are genes with the most significant change (log 2 fold change >3). *left*, from top down: Ifit1, 2010002M12Rik, Ifi44, Rsad2, Ccl7, Slfn1, Slfn4, Isg15, Mx1, Tgtp1, Gbp3, Ifit3, Ifit2, Ms4a4c, Cmpk2, Ddx60,Gm14446, Pydc4, Oas3,Oasl1, Zbp1, Oasl2,Xaf1, Apol9b, Gbp2b, Cxcl10, Irf7, BC094916, Trim30b, Pyhin1, Herc6, Ddx4, Gbp6, Slfn8; *right*, from bottom up: Oas1a, Pydc4, Oas2, Oas3, Clec4e, Oasl2. The data were calculated from average of multiple duplicate (n = 3) of mice gene expression values in each group, the gene expression data of different samples is normalized to upper quartile of fragments before calculation. Significant up—regulation (log 2 fold change >2) were also found in additional 51 genes, including Mx2, Oas1a,Oas1b, Trim30d, Stat1,Trim5 and Samhd1. See also S1 Table for details. **(D)** Fold changes of expression levels of ADARs in HDV inoculated hNTCP-Tg, hNTCP-Tg/*IFNα/βR1* ^-/-^ or WT C57/BL6 mice in comparison to that of mock—inoculated hNTCP-Tg mice. Mice were inoculated with about 5×10^10^ GEq of HDV or not, as indicated, on day 9 after birth and were sacrificed on day 6 after the inoculation. The relative mRNA levels of ADARs were determined by qRT-PCR and cellular GAPDH was used as an internal control. The average mRNA level of ADAR1L,ADAR1S, ADAR2 from three mock—inoculated hNTCP-Tg mice was set as 1.0. Results from individual mouse were shown.

We further examined HDV infection in hNTCP-Tg mice with deficiency in IFNα/βR1 (hNTCP-Tg/ *IFNα/βR1* ^-/-^), which were established by crossing hNTCP-Tg with IFNα/βR1 null mice. It’s known that IFNα/βR1 is essential for type I IFN-mediated signal transduction and its deficiency greatly reduces host antiviral activities in general [15,16]. Consistent with this notion, the efficiency of HDV infection in hNTCP-Tg/IFNα/*βR1* ^-/-^ mice was significantly higher than that in the normal hNTCP-Tg mice examined by qPCR (Fig 4A) and immunofluorescent staining of HDV delta antigen (S3A Fig),with about 10 folds increase of HDV RNA copies and 3–5 folds of more delta antigen positive cells, respectively. However, to our surprise, HDV infection was also efficiently cleared within 2 weeks in the hNTCP-Tg/*IFNα/βR1* ^-/-^ mice (n = 14) (Fig 4B), suggesting that clearance of HDV infection in the transgenic mice may also be achieved *via* type I IFN-independent mechanism(s). In order to further investigate the mechanisms of HDV clearance in the infected mice, we performed RNA—seq analysis to capture the transcriptomic landscapes in the liver of different hNTCP-Tg mouse lines upon HDV infection (n = 3 in each group). The cellular factors that mediate the type I interferon antiviral response are ISGs [17]. We first compiled a list of 583 mouse ISGs based on the previously reported datasets [18,19], and analyzed their expression fold changes in the infected mice (S1 Table). Comparing to the mock—infected hNTCP-Tg mice, hNTCP-Tg mice infected by HDV exhibited an elevated level of ISGs in the liver sample (S3B Fig). A dot plot for ISG expression fold change is shown in Fig 4C. Ifit1, Ifi44, Rsad2, Ccl7, Slfn1, Isg15, Mx1, Tgtp1, Gbp3, Ifit3, Ifit2, Ddx60, Oasl1, Zbp1, Oasl2, Cxcl10 and Irf7 were among the most significantly up—regulated ISGs in the hNTCP-Tg mice comparing to the wild—type mice similarly inoculated with HDV (Fig 4C, *left*). In marked contrast, no ISGs were significantly induced in HDV—infected hNTCP-Tg/*IFNα/βR1* ^-/-^ mice, although the virus infection was also efficiently cleared in those mice (Fig 4C, *right*). Together these results indicate that although HDV infection of hNTCP-Tg mice induces a type I IFN response, which may subsequently suppress the replication of the virus, other cellular factors may mediate IFN—independent clearance of HDV infection.

To identify cellular factors mediating IFN—independent innate immune response against HDV, a systematic investigation using hierarchical clustering analysis of total 7802 genes expressed in the livers of infected mice was performed. The transcriptome analysis revealed multiple genes were up—regulated in HDV inoculated hNTCP-Tg/*IFNα/βR1* ^-/-^ mice. Among them, 22 genes (Gm26130, mt-Ts2, Mgst2, Cyp7a1, Vps45, etc.) were clustered as a hot block next to a block containing Irf7 gene that is the master regulator for the induction of type I interferon during viral infection (Honda et al., 2005) (S4 Fig). Interestingly, most of these 22 genes apparently do not have a known function in the host anti—pathogen process or innate immunity. Together, these results unveiled the interaction landscape of HDV and the hosts, and they also indicate that multiple ISGs in hNTCP-Tg and additional novel cellular factors identified in hNTCP-Tg/ IFNα/βR1 null mice may be relevant to the clearance of HDV infection in the animals.

### HDV RNA editing occurs in infected mice

HDV RNA editing is a crucial step late in theHDV lifecycle for switching from viral RNA replication to packaging. HDV RNA editing was detected in mouse liver injected with copies of HDV DNA genome using hydrodynamics—based transfection *in vivo* [20]. It has been shown by *in vitro* studies that HDV RNA editing is controlled by ADAR1 which is also an ISG [21] [22]. Using the novel mouse models, we examined the levels of ADAR1 and HDV editing upon the viral infection *in vivo*. The expression level of both ADAR1 variants, ADAR1S and ADAR1L, was induced in hNTCP-Tg mice upon the infection (Fig 4D). Consistent with ADAR2 not being an ISG, HDV infection did not induce ADAR2 level in hNTCP-Tg mice. Intriguingly, the degree of HDV RNA editing was comparable in hNTCP-Tg/*IFNα/βR1* ^-/-^ and hNTCP-Tg mice (S5A and S5B Fig), hence it is tempting to speculate that the baseline expression of ADAR1 in these mice may be sufficient for HDV RNA editing. We further examined the degree of viral RNA editing as a function of time upon *de novo* HDV infection of hNTCP-Tg mice. The result showed the degree of viral RNA editing in the animals increased from day 6 to day 18 after infection; nonetheless, during the entire experiment period, only a small fraction of viral RNA was edited with the highest ratio of 4.6% on day 18 after viral infection (S5C Fig).

## Discussion

HDV is enveloped by glycoproteins derived from HBV [23], and it shares species restriction with its helper virus HBV at entry level. Mice are not a natural host for both HDV and HBV and do not support *de novo* infection by either virus. In this study, we demonstrated that hNTCP-Tg C57BL/6 mice can be infected by HDV. Unlike HDV, HBV infection may be restricted by unknown host factors in mice as successful HBV infection has only been achieved in hNTCP expressing hepatoma cells from human but not mouse [7,8]. It is speculated that in addition to hNTCP that facilitates HBV entry, other human hepatocyte—specific factors may be required to enable the mouse hepatocytes to support efficient formation of HBV cccDNA, which is essential for establishment of HBV infection [24,25]. Nevertheless, our work reported herein provides strong genetic evidence suggesting that hNTCP is a functional receptor for HDV infection *in vivo*.

Both human— and mouse NTCP can co—transport bile salts from circulation into hepatocytes with sodium [26]. In agreement with the results from cell—based studies suggesting that mouse Ntcp is not a functional receptor for HDV, the wild—type neonatal C57BL/6 mice which express mouse Ntcp at a level similar to that of hNTCP in the hNTCP-Tg littermates, did not support HDV infection. In contrast, mice bearing hNTCP, irrespective of the sex or the homozygosis of hNTCP transgene, were readily susceptible to HDV infection. Intriguingly, the receptor binding pre-S1 lipopeptide was shown to be able to bind to mouse hepatocyte *in vitro* [27] and *in vivo* [28], and to mouse NTCP albeit at a lower efficiency as elucidated by us [7]. The mRNA level of hNTCP was comparable to that of endogenous mNTCP in the hNTCP-Tg mice. It is unclear from current study whether the endogenous mNTCP competes for the pre-S1 domain mediated HDV interaction with hNTCP and thereby negatively affects the viral infection. Apparently mNTCP did not exert a trans—dominant negative effect on hNTCP in the transgenic mice. It will be interesting to compare HDV infection efficiency between hNTCP-Tg mice and mice with their endogenous NTCP genes replaced by hNTCP. Nonetheless, HDV effectively replicated in the liver of hNTCP transgenic mice, Northern blot analysis readily detected antigenomic RNA that is the intermediate and a diagnostic marker of HDV replication. Moreover, although quantifying the degree of RNA editing was limited by the resolution of the RNA editing assays, the study showed that HDV underwent evident RNA editing, an event essential for production of large delta antigen and switch to viral assembly, in the hNTCP-Tg mice during the *in vivo* infection. Importantly, the HDV infection of hNTCP-Tg mice could be effectively blocked by monoclonal antibodies recognizing either pre-S1 or S domain of HBV envelope proteins, suggesting that interactions between pre-S1 and hNTCP as well as S and heparan sulfate proteoglycans on hepatocytes are essential for HDV infection *in vivo*.

In contrast to immune—deficient uPA/SCID mice implanted with human hepatocytes, which have been used for studying HDV infection and drug candidate evaluation [29,30], hNTCP transgenic mice and their derivatives are heritable, easier to handle and more consistent among individual animals. They can serve as valuable and convenient models for evaluating antivirals, in particular HDV entry inhibitors. Because entry of HBV and HDV are both mediated by envelope proteins of HBV, hNTCP-Tg mice thus may also be used for evaluating HBV entry inhibitors using HDV as a surrogate. Moreover, by crossing with other mice bearing well—defined mutation(s) of various immunodeficiency and large—scale analysis of liver transcriptome, they created an unprecedented opportunity for in—depth studies of HDV viral infection and the host immune defense against HDV infection *in vivo*. In fact, our work reported herein has already revealed several unique characteristics of HDV infection in the transgenic mice.

First, we showed that neonatal but not adult hNTCP-Tg C57BL/6 mice supported readily detectable HDV infection in the liver. It is known that significant differences exist between adults and neonates in innate as well as adaptive immunity. For example, the neonatal innate immune system is biased against the production of pro—inflammatory cytokines [31] and dendritic cells (DC) may be immature until about 5 weeks of age [32]. In addition, age—dependent susceptibility of mice to virus infection has been reported for many different viruses and frequently the infection efficiency is related to the mouse genetic background [33–35]. It remains to be tested whether the HDV infection efficiency differs by age in other mouse strain(s). In addition, effects of intrahepatic immunity maturation and other age—dependent physiological changes on the susceptibility of HDV infection can also not be ruled out [36,37].

Second, concerning the infection efficiency of HDV infection in mice *in vivo*, we showed that inoculating hNTCP-Tg C57BL/6 mice at 9 days after birth with 3.3X10^10^ mge of HDV resulted in about 3% cells infected by the virus as indicated by the immunofluorescent staining of the delta antigen. It was reported that passage of HDV to woodchucks chronically infected by WHV could infect 10 to 40% hepatocyte, depending on whether the inoculated virus was first or second passage of HDV in woodchucks [38]. However, as there was no HBV infection in hNTCP-Tg mice, HDV only underwent single round infection in the mouse model reported here. The observed infection rate in the animals may also be affected by other factors, such as the route of inoculation and variations among HDV preparations. Direct intravenous injection of the virus may increase the infection rate in hNTCP-Tg mice, but it was not feasible for the 9-days animals.

Third, we observed that HDV infection of hNTCP-Tg mice was transient, irrespective to the status of their immune competency (with *Prkdc* ^scid^ or *IFNα/βR1* ^-/-^). Previous studies reported that no significant liver histopathological changes were found in HDV transgenic mice [39,40] or in experimental HDV infected chimpanzees [41]. Reports on experimental HDV inoculation into neonatal or SCID mice with WHV enveloped HDV, which took advantage of HDV ribonucleoprotein’s compatibility with WHV envelops thereby bypassing the species restriction at entry level of HDV, showed a transient infection in the livers of infected animals [42]. We showed herein that the receptor mediated, *de novo* infection of HDV was cleared in about two weeks in the hNTCP-Tg mice upon viral inoculation with no obvious liver pathological changes. Interestingly, only few cells positive for HDV delta antigen were found to be TUNEL positive in the liver samples of infected mice. More studies, ideally using hNTCP-Tg mice deficient in hepatic apoptosis or necrosis, are needed to clarify whether HDV infection results in the death of the hepatocytes in the mice. Surprisingly, intrahepatic HDV RNA was cleared after infection at comparable kinetics among normal, homozygous *Prdkc* ^*scid*^ and *IFNα/βR1* ^-/-^ hNTCP-Tg mice; this suggested that the clearance of HDV infection in hNTCP-Tg mice was either due to the activation of innate immune response or accumulation of large delta antigen that suppressed HDV RNA replication. However, the latter hypothesis is not supported by a recent finding that HDV mono—infection of immune deficient (SCID/beige) mice transplanted with human hepatocytes persisted intrahepatically for more than 6 weeks [29]. It will be interesting to further investigate the underlying mechanisms controlling the apparently different outcome of the HDV infection in the hNTCP transgenic versus the human hepatocyte—transplanted mouse models, for example whether the difference is due to the activity of NK cells presented in the hNTCP-Tg/*Prdkc* ^*scid*^ but not in the xenotransplanted SCID/beige mice. Another possible explanation of the discrepancy between the two models is that HDV infection may induce production of cytokines or other soluble factors by non—parenchymal cells that species—specifically inhibit HDV replication in hepatocytes of the infected mice.

Forth, two lines of independent evidence presented in this study strongly suggest that HDV infection of hNTCP-Tg mice induces a type I IFN response suppressing HDV replication in hepatocytes. Firstly, intrahepatic HDV RNA in IFNα/βR1 null hNTCP-Tg mice is about 10-fold higher than that in normal hNTCP-Tg mice. In addition, dozens ISGs, among which some have been characterized for their antiviral activities against various other viral infections, for example Mx1, Ifit1, Isg15, Ifi44, Ddx60, Oasl (Liu et al., 2012; Schoggins et al., 2011) and Irf7 were up-regulated upon HDV inoculation in hNTCP-Tg mice. This is the first report demonstrating that HDV infection induces an early activation of IFN response *in vivo*. Of note, Hartwig et al. showed that treatment of cultured cells with IFNα increased both ADAR expression levels and RNA editing [43] and enhanced HDV RNA editing was shown to increase the expression level of large delta antigen which could further restrict the HDV replication in cells [44]. It will be interesting to further investigate how the type I IFN response restricts HDV replication in hepatocytes *in vivo*.

Finally, liver RNA-seq analyses of HDV-infected normal and IFNα/βR1 null hNTCP-Tg mice also revealed that expression of additional cellular genes was associated with HDV infection. The majority of these genes have not been characterized for activity in immune response or antiviral infection. Interestingly, 22 genes including Gm26130, a snoRNA gene, and Cyp7a1, the rate-limiting enzyme in the synthesis of bile acid from cholesterol *via* the classic pathway, and several mitochondrial tRNAs are clustered in the analysis of 7802 liver genes of the infected mice. Although further experiments are needed to dissect the possible antiviral roles of these molecules, it is tempting to speculate that at least some of them may function in parallel or in addition to the known ISGs, and be relevant in HDV viral clearance. Of note, as the smallest virus known to infect humans, HDV encodes only one protein (delta antigen), which modulates viral replication through interaction with cellular DNA-dependent RNA polymerases and other host factors [1,2]. Studying of HDV infection in hNTCP-Tg mouse model thus opens a unique door for understanding how an animal reacts to invasion by the smallest viral pathogen.

In summary, our studies of HDV infection in hNTCP-Tg mice not only proved that NTCP is a functional receptor for HDV infection *in vivo* and hNTCP-Tg mouse is a useful model for studying antivirals against the infection, and they also shed new light on the interaction between HDV and host immunity, and laid a foundation for future investigation toward better understanding the pathogenesis of HDV infection.

## Materials and Methods

### Ethics statement

All animals were housed in the animal facility of the National Institute of Biological Sciences (NIBS), Beijing. Animal experiments were conducted following the National Guidelines for Housing and Care of Laboratory Animals and performed in accordance with NIBS institutional regulations after approved by the institution's Institutional Animal Care and Use Committee (IACUC). The protocol number is NIBS-0012.

### Production of hNTCP transgenic mice

The human NTCP gene with a C9 tag [6] was cloned into a vector with an expression cassette driven by mouse albumin enhancer/promoter. The recombinant plasmid was linearized and introduced into the pronuclei of C57BL/6NCrlVr mouse zygotes. PCR primers for identifying hNTCP transgene are hNTCP-F (5′- GGATAGGGATCCGCCACCATGGAGGCCCACAACGCG-3′) and hNTCP-BGH-R (5′-ATTTCCCTCGA GCCATAGAGCCCACCGCAT-3′).

### Mutant mice with severe combined immune deficiency or interferon (alpha and beta) receptor 1 knockout

Fox Chase SCID^®^ (CB17/Icr-Prkdc^scid^/IcrlcoCrlVr, homozygous for the severe combined immune deficiency spontaneous mutation Prkdc) mice were from the Vital River, Beijing, China; Interferon (alpha and beta) receptor 1 knockout (B6.129S2-*Ifnar1* ^*tm1Agt*^/Mmjax) mice [15] backcrossed to C57BL/6 for at least 5 generations were from the Jackson Laboratory, Maine, USA. hNTCP transgenic mice homogeneous for *Prkdc* mutation or null for IFNα/βR1 were obtained by cross breed hNTCP transgenic mice with the corresponding immune deficient mice, respectively. The genotypes of the mice were determined by PCR with DNA isolated from mouse tail. Animals were hosted in an SPF mouse facility and all animal experiments were conducted following the national guidelines for housing and care of laboratory animals and performed in accordance with institutional regulations after approval by the IACUC at National Institute of Biological Sciences, Beijing.

### Expression analysis of the hNTCP transgene

RNA from mouse liver or kidney was extracted using TRIzol^®^ Reagent (Invitrogen). The total RNA was reverse transcribed into cDNA with PrimeScript^TM^ RT-PCR Kit (Takara), cDNA obtained from 20ng RNA was used for real time PCR assay. See S1 Text. Supplemental experimental procedures for details. Western blot analysis for the expression of hNTCP was performed by using liver samples collected from hNTCP transgenic or wild-type mice, total 30 μg protein was loaded and 10μg/ml 1D4 antibody (Santa Cruz Biotech) was used for detecting the C9 tag fused at the C-terminus of the hNTCP transgene. Mouse GAPDH was used as a loading control.

### HDV production, mouse viral inoculation, quantification of HDV RNA and ADAR mRNAs in mouse liver samples

HDV was produced as previously described by using two plasmids transfection in Huh-7 cells [6]. Mice were inoculated with purified HDV by intraperitoneal (i.p.) injection. To minimize the influence of variables, in each experiment (usually presented as one panel of a figure in the manuscript), mouse littermates were injected with HDV from same viral preparation. Mouse liver samples were homogenized in liquid nitrogen immediately after collection, and then lysed by TRIzol^®^ reagent. The total RNA was reverse transcribed into cDNA with PrimeScript^TM^ RT-PCR Kit (Takara), cDNA from 20ng RNA was used for real time PCR assay. See S1 Text. Supplemental experimental procedures for details.

### Analysis of HDV genome and antigenome by Northern blot and HDV editing efficiency by RT-PCR amplification combined with Nco I digestion

Liver tissue RNA was extracted using TRIzol^®^ reagent. 2μg total RNA was electrophoresed through formaldehyde-containing 1% agarose gels, blotted onto a nitrocellulose membrane (Hybond-C Extra, Amersham), and hybridized with digoxigenin (DIG)-labeled RNA probes for HDV genome, HDV antigenome, or mouse GAPDH, respectively. For detecting HDV RNA editing, *Nco* I restriction digestion of PCR-amplified cDNA derived from HDV RNA was used. 300 ng total RNA was reverse transcribed using random hexamers, followed by PCR using primers specific to HDV cDNAs. PCR products were purified and subjected to overnight digestion with restriction enzyme *Nco* I (NEB). The total digestion products were separated by 4% polyacrylamide gel electrophoresis (PAGE), and the gel was stained with silver nitrate. In independent experiments, HDV RNA editing was also examined using the [^32^P] dCTP labeling method as reported by Casey *et al* [45] or quantified by microcapillary electrophoresis analysis using an Agilent 2100 bioanalyzer. See S1 Text. Supplemental experimental procedures for details.

### RNA-seq and data analysis

RNA-seq analysis was conducted using the total RNA of livers from 3 mock-inoculated hNTCP transgenic (hNTCP^+/-^) mice, and HDV-inoculated mice including three hNTCP^+/-^, three hNTCP^+/-^/*IFNα/βR*-^/-^, and three wild-type mice. Mice were inoculated on day 9 after birth, and sacrificed 6 days after the inoculation. RNA-seq was performed using Illumina Genome Analyzer IIx system. Sequence data was deposited at Sequence Read Archive (SRA) of the NCBI under BioProject PRJNA236433. See S1 Text. Supplemental experimental procedures for details.

## Supporting Information

S1 Table List of all compiled mouse ISGs with log2 ratio of HDV-inoculated hNTCP-Tg (or hNTCP-Tg /IFNα/βR1 ^-/-^ mice) to (HDV-inoculated wild-type mice or) mock-inoculated hNTCP-Tg mice. (XLSX)

S1 Text Supplemental experimental procedures. (DOCX)

S1 Fig Hematoxylin and Eosin (H & E), HDV delta antigens and TUNEL staining of liver sections from mice after HDV infection. (TIF)

S2 Fig HDV infection of hNTCP-Tg mice of different age. (TIF)

S3 Fig ISGs were induced upon HDV infection of hNTCP-Tg mice. (TIF)

S4 Fig Heat map of genes highly regulated in HDV inoculated hNTCP-Tg and hNTCP-Tg/ IFNa/ßR1 ^-/-^ mice. (TIF)

S5 Fig HDV underwent RNA editing *in vivo*. (TIF)
