# Supplementary material for: Hepatitis D Virus Infection of Mice Expressing Human Sodium Taurocholate Co-transporting Polypeptide
Source: PLoS Pathog. 2015 Apr 22;11(4):e1004840. doi: 10.1371/journal.ppat.1004840 (PMC4406467; doi:10.1371/journal.ppat.1004840)
Supplement: S1 Text — (DOCX) [file ppat.1004840.s002.docx]

**Supplemental Information**

**Human sodium taurocholate co-transporting polypeptide transgenic mice support**

**Hepatitis D virus infection**

Wenhui He, Bijie Ren, Fengfeng Mao, Zhiyi Jing, Yunfei Li, Yang Liu, Bo Peng, Huan Yan, Yonghe Qi,

Yinyan Sun, Jianhua Sui, Fengchao Wang, Wenhui Li

**Supplemental Experimental Procedures**

*Antibodies and other reagents*

Murine monoclonal antibodies (mAb) used in this study have been described previously [[3](#_ENREF_3)]. In brief, 2D3 specifically recognizes aa 19-36 of the pre-S1 domain of HBV L protein (Genotype D), 17B9 targets to the S domain of the HBV envelope proteins, both are able to neutralize HBV and HDV infection in cell cultures. Fluorescein isothiocyanate (FITC)-labeled 4G5 is a mcAb specific for the HDV delta antigens; 1D4 is a mcAb targeting the C9 tag with amino acid sequence TETSQVAPA. Secondary antibodies for immunofluorescence staining or Western blot were from Life Technologies (USA) or Sigma-Aldrich (USA). Quantitative real time PCR kit and reverse transcriptase reagents were purchased from Takara Inc. (Beijing, China).

*Southern blot analysis*

Mouse tail samples were lysed using a lysis buffer (50 mM Tris-HCl, pH 7.4, 10 mM EDTA, 150 mM NaCl, 1% SDS), and the genomic DNA were extracted by the standard phenol-chloroform extraction protocol. 5µg genomic DNA were digested by *Bam* HI-HF (NEB) overnight at 37℃ and then separated by 1% agarose gel. After denaturation by denature buffer (0.5M NaOH, 1.5M NaCl) and neutralization, the DNA in the agarose gel was transferred to a nylon membrane (Hybond-N^+^, Amersham), using a standard neutral transfer procedure. The nylon membrane was hybridized with a [α-32P] dCTP-labeled probe containing a 428bp chimeric fragment from 3’ hNTCP to BGH poly A region. The probe was prepared by random primer DNA labeling kit (Ver.2.0; Takara) with the hNTCP plasmid used for pronuclear injection. The membrane was incubated with 7 ml Perfect^TM^ Hyb Plus Hybridization Buffer (Sigma) for 1 hour at 67℃ for pre-hybridization, then the probe was added for hybridization at 67℃ overnight. The membrane was then washed once with 2×SSC/0.1% SDS, 1×SSC/0.1% SDS, and 0.5×SSC/0.1% SDS at 67℃ for 20 min, respectively. Finally, the membrane was subjected to autoradiographic exposure.

*Expression analysis of the hNTCP transgene*

RNA from mouse liver or kidney was extracted using TRIzol^®^ Reagent (Invitrogen) following the manufacturer’s instructions. The total RNA was reverse transcribed into cDNA with PrimeScript^TM^ RT-PCR Kit (Takara) and cDNA obtained from 20ng RNA was used for real time PCR assay. Primers for quantifying hNTCP transgene are located at the 891-913 bp and 1020-1036 bp of the human NTCP CDS, with forward primer (5′-GCTTCTCCTCATTGCCATATTTT-3′) and backward primer (5′-GGGAGCAGTCCTCCCCT-3′). The hNTCP mRNA copy numbers were calculated using a standard curve with cellular GAPDH cDNA as an internal control. Mouse Ntcp expression was quantified using forward primer (5′-GACACCACACTTACTGGCT-3′) and backward primer (5′-ATGGTGCGTCTGCAGCTTGG-3′) following the same procedure as for hNTCP. The primers were validated for their selectivity and specificity for amplification of hNTCP transgene or mouse Ntcp, respectively. The detection limits for hNTCP and mNTCP RNA are ~10 copies per 20 nanogram total liver RNA.

*HDV production and mouse viral inoculation*

HDV was produced as previously described by transfecting two plasmids in Huh-7 cells [[3](#_ENREF_3)]. In brief, pCMV-HDV3.0 containing a head-to-tail trimer of 1.0×HDV cDNA (Genotype I, AF425644.1) and pUC-HBV-LMS plasmid (containing nucleotide 2431~1990 of HBV, U95551.1) were transfected into Huh-7 cells, the cell culture supernatant was collected and HDV was precipitated by 8% PEG8000. The precipitation was suspended in PBS and purified with ultra-centrifugation at 4℃, 32000 rpm (MLS50, Beckman) for 9h. The viral pellet was re-suspended in PBS, viral RNA genome equivalents (GEq) was determined by qRT-PCR and aliquot was frozen at -80℃ until use. Mice were subcutaneously (s.c.) injected with dexamethasone sodium phosphate at a dose of 0.05 mg/g body weight and were challenged with purified HDV by intraperitoneal (i.p.) inoculation. The mouse weight varied depending on the age, litter size and other factors. The weight of 9- day mice was around 5g; 15g (male) or 12.5(female) on 28 days; 22g (male) and 18g (female) on day 49 after birth, the dose of viral inoculum was adjusted to the weight of mice accordingly. An HDV inoculating dose range from 1.6-6.7×10^10^GEq per mouse of 9-day old was used for imaging, Northern blot, RNA editing, RNA-seq and antibody neutralization experiments depending on the availability of the mice and virus; a lower dose of 2×10^9^GEq HDV/g (about 1×10^10^GEq per mouse of 9-day old) inoculation was used for kinetic studies of viral infection and clearance unless otherwise indicated. In the immunofluorescence staining study shown in Fig.2B, 3.3×10^10^GEq HDV were i.p. inoculated into each 9-day mouse with an estimated 4.6×10^7^ liver cells, assuming all the inoculated HDV could reach the mouse liver, this approximately equaled to 717 multiplicities of genome equivalents (mge). It is difficult to directly compare the infectivity of HDV among different research groups due to different genotype and/or origin of HDV (serum or transfection derived). HDV used in this study infected 10~30% HepG2-NTCP cells when inoculated for 16 hours with ~5% PEG8000 at 500 mge (5×10^8^GEq HDV/1×10^6^ cell ) in cell culture.

*Quantification of HDV RNA and ADAR mRNAs in mouse liver samples*

Mouse liver samples were homogenized in liquid nitrogen immediately after collection, and then lysed by TRIzol^®^ reagent following the manufacturer’s instructions. The total RNA was reverse transcribed into cDNA with Prime^TM^ Script RT-PCR Kit (Takara), cDNA obtained from 20 ng RNA was used for real time PCR assay. Primers for quantifying HDV total RNA or genome equivalent copies are located in the delta antigen coding region of HDV RNA genome with sequence: forward primer HDV-1184F (5′-TCTTCCTCGGTCAACCTCTT-3′), and backward primer HDV-1307R (5′-ACAAGGAGAGGCA GGATCAC-3′). Real time PCR was performed on an ABI Fast 7500 real time system instrument (Applied Biosystems, USA). The detection limit for HDV RNA is 10~100 copies per 20 nanogram total liver RNA. Primers for quantifying ADAR mRNA are: ADAR1L forward primer (5′-TCTCAAGGGTTCAGGGGACC-3′) and backward primer (5′-GCTGCGGGTATCTCCACTTG-3′); ADAR1s forward primer (5′-GTTTCGCAGAGG TAACCCCA-3′) and backward primer, (5′-TTGGATGTGCATCTGGGGA-3′); ADAR2 forward primer (5′-TCCTGCAGTGACAAGATAGCA-3′) and backward primer (5′-GGTTCCACGAAAATGCTGAG-3′). The HDV viral genome equivalent copies or the mRNA level of mouse ADARs were calculated with a standard curve and the cellular GAPDH RNA was used as an internal control.

*Northern blot analysis of HDV genome and antigenome*

Liver tissue RNA was extracted from hNTCP transgenic mice challenged with HDV using TRIzol^®^ Reagent (Invitrogen). 2µg total RNA was treated with formaldehyde, electrophoresed through formaldehyde- containing 1% agarose gels, blotted onto a nitrocellulose membrane (Hybond-C Extra, Amersham), and hybridized at 68℃ with 1008 nt dig-labeled HDV genome specific probe, complement to 260 nt to 1267 nt of the HDV genome sequence (from 5' to 3'); or, 1008 nt HDV antigenome probe, complement to 408 nt to 1415 nt of the HDV antigenome sequence (from 5' to 3'); or, mouse GAPDH probe. HDV probes and mouse GAPDH probe were transcribed and labeled *in vitro* from T7 promoter with the Roche DIG Northern Starter kit (Roche), mouse GAPDH probe was used as a loading control.

*Analysis of HDV editing efficiency by RT-PCR amplification combined with Nco I digestion and silver staining or radiolabeling with [α-32P] dCTP*, *or microcapillary electrophoresis using the Agilent 2100 bioanalyzer*

Analysis of HDV RNA editing was based on a published assay [[4](#_ENREF_4),[5](#_ENREF_5)] with modifications. In brief, liver tissue RNA was extracted from hNTCP transgenic mice challenged with HDV using TRIzol^®^ reagent (Invitrogen). About 300 ng total RNA was used as template for reverse transcription at 37℃ for 15 min, using random hexamers (Takara) as primers. The cDNA was amplified by 37 cycles of PCR using primer HDV-edit-F (5’-GAGATGCCATGCCGACCCGAAGAG-3’) and HDV-edit-R (5’-CAGCAGTCTCCTCTTTACAGA-3’). The PCR amplifies a 776bp fragment covering the specific HDV RNA editing (Amber/W) site corresponding to a DNA sequence of CCAT**A/(G)**G. PCR products were purified with PCR purification kit (Takara) followed by overnight digestion with restriction enzyme *Nco* I (NEB). The total digestion products were separated by 4% polyacrylamide gel electrophoresis (PAGE), then treated with fix buffer (10% acetic acid) for 10 minutes. After washing with H_2_O twice, the gel was stained with a mixture of staining buffer (1g/L AgNO3 , 1.5ml/L 37% formaldehyde) for 15 minutes. Following washed for 5~10 seconds, the band was developed with developing buffer (30g/L NaOH, 4ml/L 37% formaldehyde). HDV RNA editing is indicated by the appearance of a 648bp band after *Nco* I cutting of the amplification product. In an independent experiment using [α-32P] dCTP radiolabeling method, we performed the reverse transcription and PCR using the protocol and primers as described above. Then 300 ng gel-purified PCR products were added to a 50µl single-cycle PCR radiolabeling mixture. The single-cycle PCR system contained 10 µCi of [α-32P] dCTP (3000 µCi/mmol; Amersham) and 3.3 nmol each dATP, dTTP and dGTP in a 50 µl PCR system, the PCR was conducted as following: 3min at 98℃, 1min at 65℃ and 3 min at 72℃. After purification, the PCR products were digested with *Nco* I overnight, followed by polyacrylamide electrophoresis and autoradiography. For microcapillary electrophoresis assay, the *Nco* I digested DNA was purified with PCR product purification kit (Qiagen) and eluted in 10µl elution buffer. The DNA concentration was determined using The Qubit® dsDNA HS (High Sensitivity) Assay Kit (Invitrogen) and 500pg DNA was analyzed by microcapillary electrophoresis using an Agilent 2100 bioanalyzer (Agilent). Agilent 2100 Bioanalyzer applies a chip technology to perform microcapillary electrophoresis, DNA is separated analogously to a capillary electrophoresis and normalized to the ladder DNA, according to the size and molarity concentrations of the ladder DNA. The size and concentration of the unedited 776bp and edited 648bp DNA was analyzed by the Agilent Expert 2100 Software. The RNA editing ratios were calculated based on the molarity concentrations of these two DNA fragments. The reannealing reactions after PCR amplification generate heteroduplexes, which are resistant to *Nco* I digestion. Thus, in the assays of silver staining and microcapillary electrophoresis of *Nco* I digested products, the undigested heteroduplexes will give an underestimate of amount of editing. While detection of *Nco* I digested products from amplificons radioactively labeled with [α-32P] dCTP in an additional step of single-cycle PCR helps to avoid this pitfall.

*Immunofluorecent staining*

Mouse liver tissue was fixed in 4% paraformaldehyde immediately after dissection, and dehydrated in 30% sucrose solution for 8 hours before embedded in OCT for frozen sectioning, followed by cutting as thin sections (6 µm) and mounting on glass slides. Sodium borohydride solution (Sigma, 1mg/ml) and citrate buffer solution (pH=6.0) was applied on the slides to retrieve antigen. Tissue sections were then treated with 0.5% Triton-X100 for 30 minutes for permeabilization. Bovine serum albumin solution (3% in PBS) was used to block the unspecific binding. HDV Delta antigens were stained by FITC-labeled 4G5 antibody for 30 minutes in 37℃ and then washed with PBS. Slides were mounted by mounting solutions containing 1µg/ml 4',6-diamidino-2-phenylindole (DAPI) and images were acquired by a Zeiss LSM510 Meta laser scanning confocal microscope or a Nicon A1R+ laser scanning confocal microscope.

*TUNEL staining*

Mouse liver slides were prepared as that used for immunofluorescent staining. TUNEL staining was conducted using an In Situ Cell Death Detection Kit (Roche) following the kit’s instruction. In brief, after permeabilization with 0.5% Triton-X 100, slides were applied with TUNEL reaction mixture, and incubated for 60 minutes at 37℃, protected from light. Then the slides were washed with PBS. Positive controls were treated with DNase (RQ1 RNase-Free DNase,Promega) after permeabilization. Slides were mounted with ProLong® Gold Antifade Mountant (Life Technologies) and images were recorded.

*Hematoxylin and Eosin (H&E) staining of liver sections from mice inoculated with HDV*

H&E staining of mouse liver was conducted following standard protocol. In brief, liver tissues were fixed in 4% paraformaldehyde overnight and embedded in paraffin. 5 µm liver tissue sections were deparaffinized and rehydrated, followed by staining with hematoxylin for 5 minutes and counterstaining for 30 seconds in eosin solution.

*RNA- seq and data analysis*

Three hNTCP transgenic (hNTCP^+/-^) mice, 3 hNTCP^+/-^ /IFNα/βR1 ^-/-^ mice, and 3 wild-type mice were inoculated with 1.6×10^10^ GEq HDV, respectively on day 9 after birth. Three 9-day old hNTCP^+/-^ mice were mock-inoculated. The mice were sacrificed 6 days post inoculation and liver RNA was extracted using TRIzol^®^ reagent (Invitrogen). Libraries were constructed with Illumina mRNA-Seq Sample Prep Kit. A 42bp single-end sequencing was performed using Illumina Genome Analyzer IIx and Sequencing Kits. Reads were mapped to the mouse reference genome (release GRCm38) with Bowtie [[6](#_ENREF_6)]. Assembly, abundances estimation and differential expression were calculated with Cufflinks 2.1.1 [[7](#_ENREF_7)] using a known set of reference transcripts from Ensembl v.73. To conduct a large-scale mouse antiviral ISG analysis, lately published compilation of ISGs in human [[2](#_ENREF_2)] was used as a source for registering their mouse homolog. By combining the available datasets of the mouse ISGs and mouse homologs of human ISGs [[1](#_ENREF_1)], we compiled a list of 583 mouse ISGs and plotted their expression fold changed in HDV infected hNTCP-Tg to mock-inoculated hNTCP-Tg mice, HDV infected hNTCP-Tg mice to HDV inoculated wild-type mice and HDV inoculated hNTCP^+/-^/IFNα/βR1^-/-^ mice to HDV inoculated wild-type mice. All dot plots and heat maps were generated with R (RDC Team, 2011). Gene expression high throughput sequencing data used to analyze changes of gene expression after HDV infection was deposited at Sequence Read Archive (SRA) of the NCBI under BioProject PRJNA236433.

**Supplemental References**

1. Liu SY, Sanchez DJ, Aliyari R, Lu S, Cheng G (2012) Systematic identification of type I and type II interferon-induced antiviral factors. Proc Natl Acad Sci U S A 109: 4239-4244.

2. Schoggins JW, Wilson SJ, Panis M, Murphy MY, Jones CT, et al. (2011) A diverse range of gene products are effectors of the type I interferon antiviral response. Nature 472: 481-485.

3. Yan H, Zhong G, Xu G, He W, Jing Z, et al. (2012) Sodium taurocholate cotransporting polypeptide is a functional receptor for human hepatitis B and D virus. Elife 1: e00049.

4. Casey JL, Gerin JL (1995) Hepatitis D virus RNA editing: specific modification of adenosine in the antigenomic RNA. J Virol 69: 7593-7600.

5. Polson AG, Ley HL, 3rd, Bass BL, Casey JL (1998) Hepatitis delta virus RNA editing is highly specific for the amber/W site and is suppressed by hepatitis delta antigen. Mol Cell Biol 18: 1919-1926.

6. Langmead B, Trapnell C, Pop M, Salzberg SL (2009) Ultrafast and memory-efficient alignment of short DNA sequences to the human genome. Genome Biol 10: R25.

7. Trapnell C, Hendrickson DG, Sauvageau M, Goff L, Rinn JL, et al. (2013) Differential analysis of gene regulation at transcript resolution with RNA-seq. Nat Biotech 31: 46-53.
