# Supplementary figures and images for: Hepatitis D Virus Infection of Mice Expressing Human Sodium Taurocholate Co-transporting Polypeptide
Source: PLoS Pathog. 2015 Apr 22;11(4):e1004840. doi: 10.1371/journal.ppat.1004840 (PMC4406467; doi:10.1371/journal.ppat.1004840)

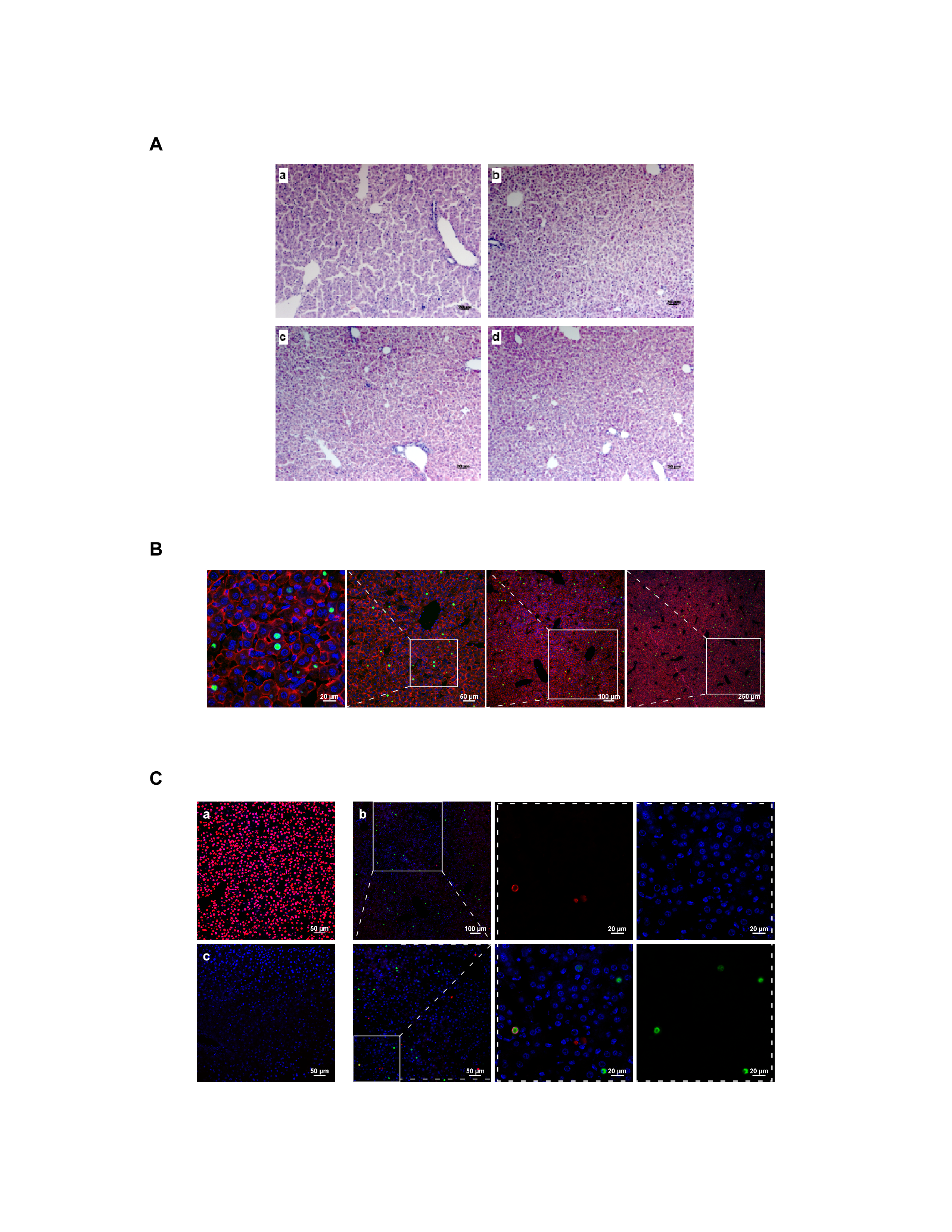

Supplement: S1 Fig — (TIF) [file ppat.1004840.s003.tif]

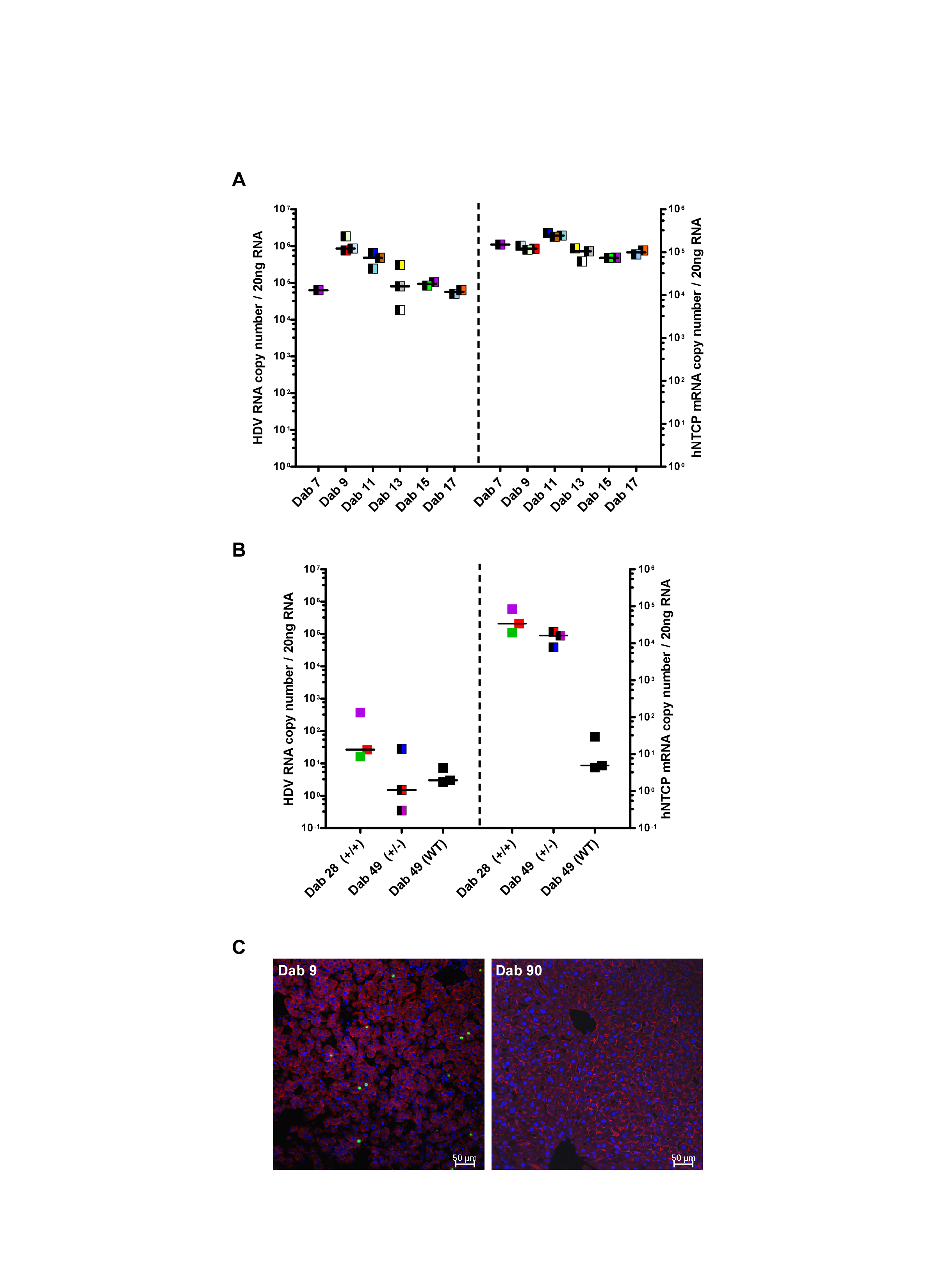

Supplement: S2 Fig — (TIF) [file ppat.1004840.s004.tif]

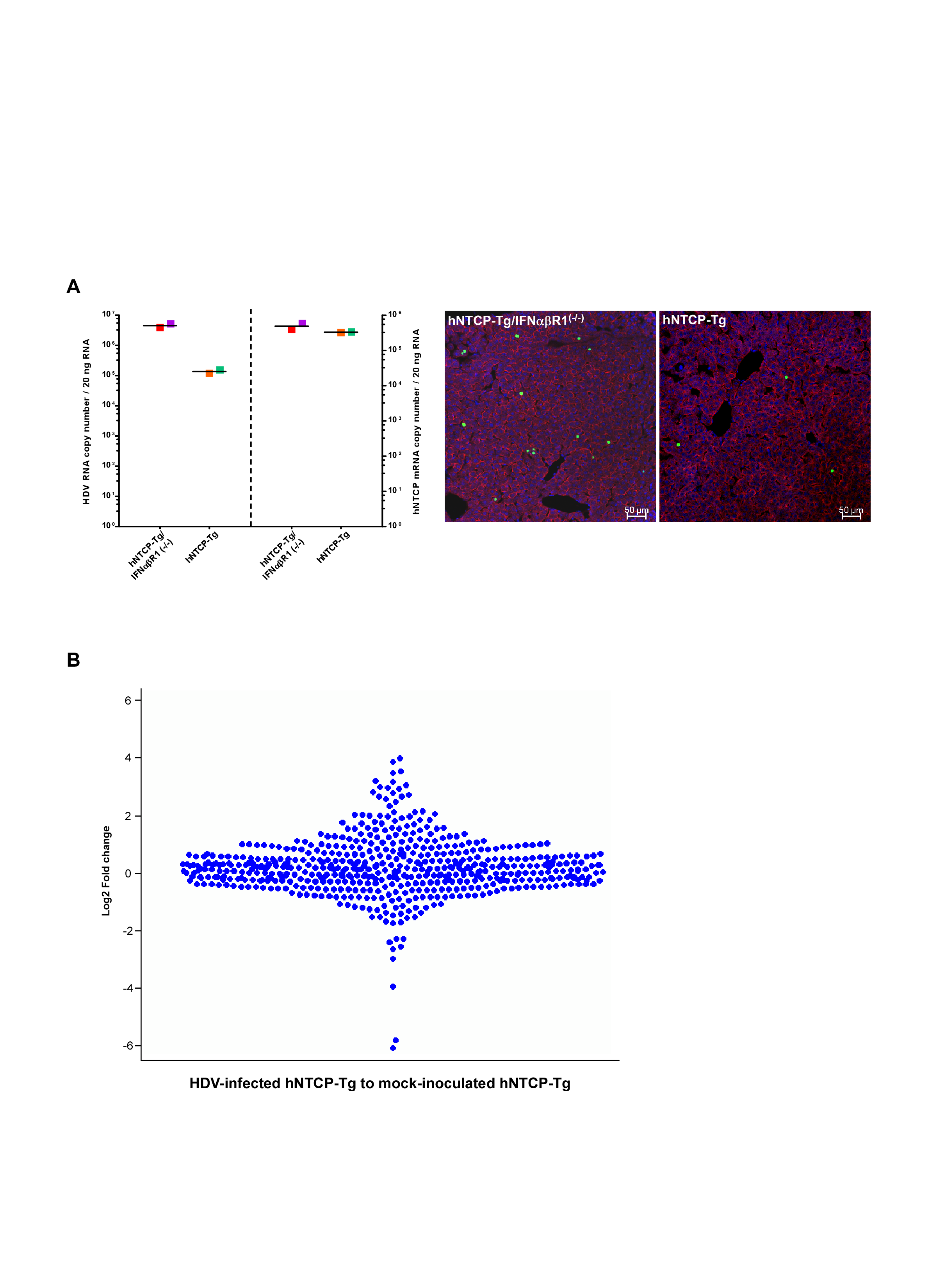

Supplement: S3 Fig — (TIF) [file ppat.1004840.s005.tif]

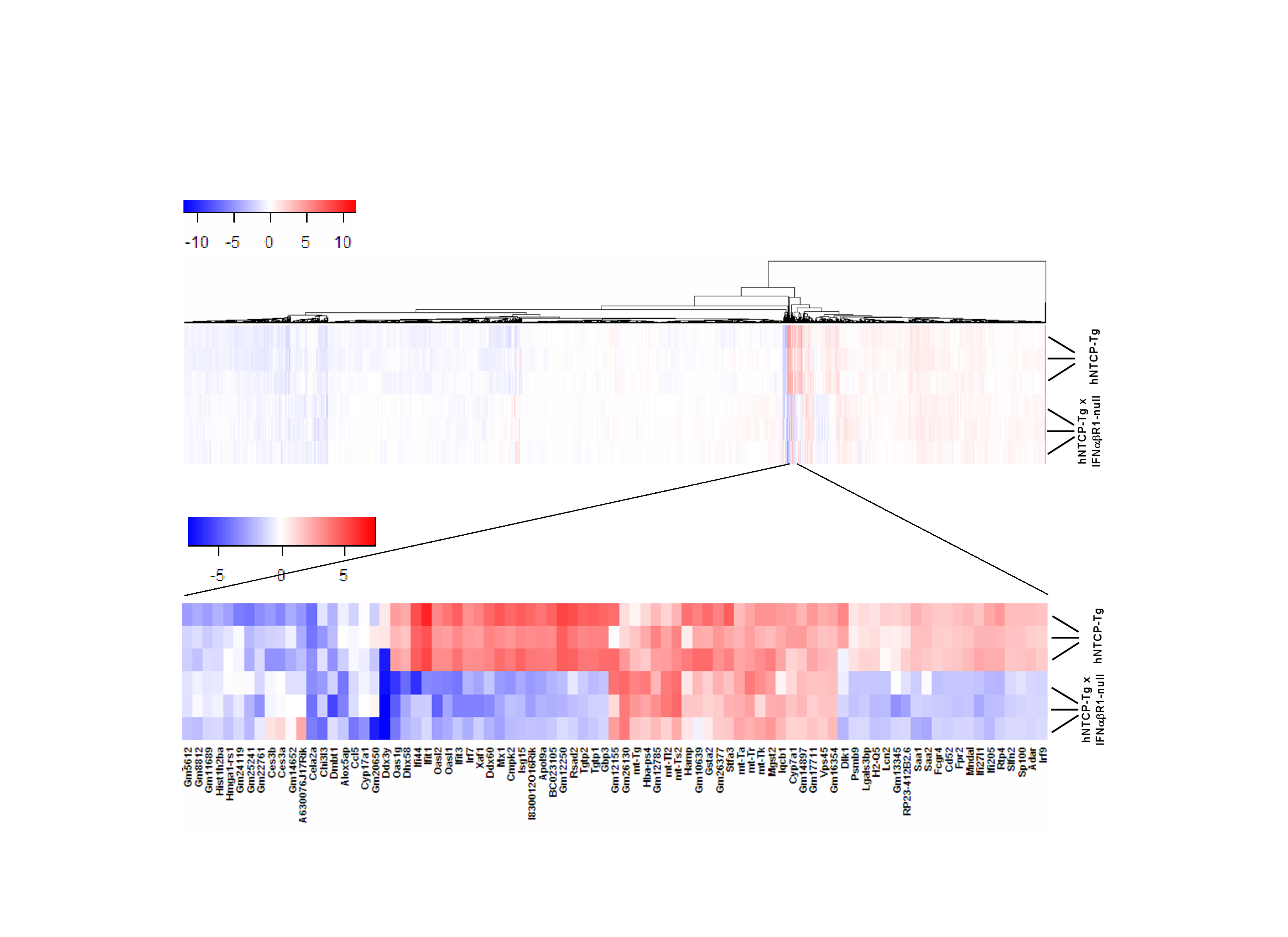

Supplement: S4 Fig — (TIF) [file ppat.1004840.s006.tif]

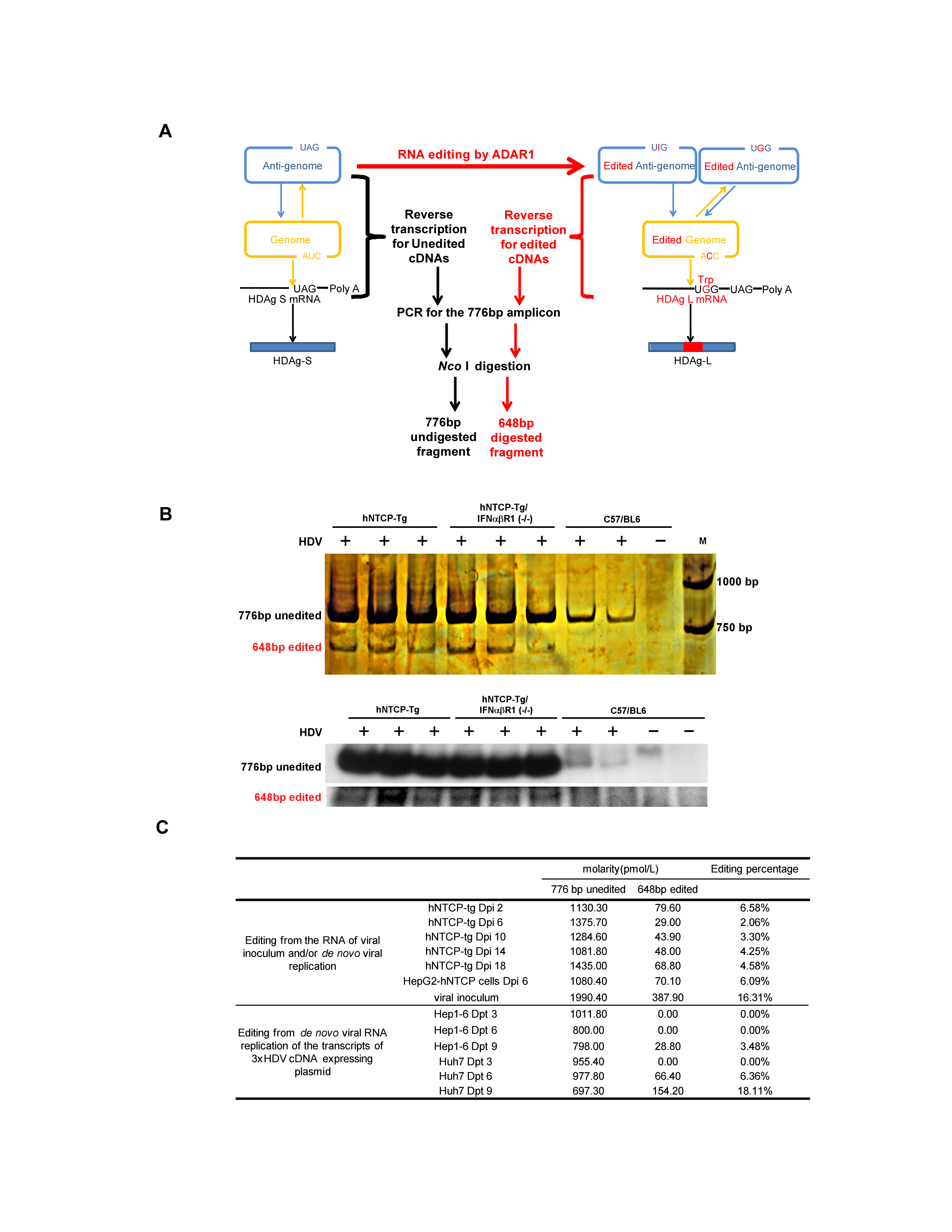

Supplement: S5 Fig — (TIF) [file ppat.1004840.s007.tif]
